# Improvement of Pain Management by Nefopam in a Rat Adjuvant-Induced Arthritis Model

**DOI:** 10.3389/fvets.2022.809980

**Published:** 2022-04-26

**Authors:** Elisa Dalix, Mathieu Maalouf, Marie-Thérèse Linossier, Arnaud Vanden-Bossche, Sylvie Peyroche, Hubert Marotte

**Affiliations:** ^1^INSERM, U1059-SAINBIOSE, Université de Lyon, Saint-Etienne, France; ^2^Department of Rheumatology, Hôpital Nord, University Hospital Saint-Etienne, Saint-Etienne, France

**Keywords:** animal welfare, pain management, nefopam, arthritis, inflammation

## Abstract

**Introduction:**

The adjuvant-induced arthritis (AIA) model is widely used in research to investigate arthritis pathogenesis. Hind paw inflammation is the main outcome in this model with high loss of mobility function partly related to pain. However, analgesics such as non-steroidal anti-inflammatory drugs or opioid drugs interfere with the inflammation process related to arthritis, thus reducing their beneficial use in this model. Therefore, we investigated the effect of nefopam on arthritis development in order to improve pain management in the AIA model.

**Methods:**

Female Lewis rats were randomly divided into two groups, and each group received an injection of *Mycobacterium butyricum* on defining day (D) 0. At D6, rats (*n* = 10) received nefopam (intraperitoneally or orally) or NaCl 0.9% IP or 1% sucrose in water (*n* = 5 for each). Rats were monitored with the arthritic index (AI) and ankle circumference. Pain was assessed by scoring based on behavioral indicators. Histology, RT-qPCR, and microcomputed tomography were performed.

**Results:**

The clinical parameter AI and ankle circumference were not different in both groups at various time points. However, pain score was significantly lower in the nefopam group at the early stage of the disease. At a later stage of the disease, inflammation was mildly lower whereas bone erosion and bone loss parameters increased in the nefopam group.

**Conclusion:**

Nefopam provided a slight reduction in the level of pain at the arthritis onset without reducing arthritis severity and bone loss in the rat AIA model. However, it should be administrated orally for a shorter period to avoid inflammation reduction in the long run.

## Introduction

The adjuvant-induced arthritis (AIA) is a well-known model used to investigate arthritic diseases for their pathophysiologic similarities to human arthritis. This model is widely used to investigate arthritic diseases (1–3). This is a robust model with rapid arthritis onset, high arthritis prevalence, and easy clinical measurements (4). The AIA model develops arthritis with inflammation of the hind paws and high bone resorption, especially at the navicular bone (5). Inflammation plays a key role in disease development, so the commonly used analgesics, such as non-steroidal anti-inflammatory drugs (NSAID), not only reduced the pain but also inflammation in this model (6). Similarly, opioid drugs reduce pain and inflammation in this model (7). As this model is essential for the investigation of arthritic diseases, we attempted to improve pain management in this model. Nefopam is an antinociceptive compound, part of benzoxazocine class drugs, which acts centrally at spinal and supra-spinal sites (8). Therefore, it acts through different mechanisms of action compared to NSAID or opioids (9). Even though its mechanism of action is not fully understood, nefopam seems to be involved in the inhibition of reuptake of monoamines (norepinephrine, serotonin, and dopamine) in the central nervous system. This molecule is not widely used in animal experimentation, but it already demonstrated antalgic effects on rats (8). Antinociceptive responses in the acute pain model were observed at doses of 10–30 mg/kg intraperitoneally (IP) or subcutaneously (SC) and 60 mg/kg orally [*per os* (PO)] (8). The aim of this study was to improve pain management in the AIA model by nefopam administration without interfering with arthritis development.

## Methods

### Animals

This study was approved by the Ethical Committee for Animal Experiments of Saint-Etienne University (agreement number: 2019032816186046). Experiments were conducted using 6-week old Lewis female rats (Charles River Laboratories, L'arbresle, France). Animals were housed with a 12 h light/dark cycle and with *ad libitum* food and water access. *Mycobacterium butyricum* (Difco Laboratories, Detroit, MI, US) dissolved in 200 μl sterile mineral oil (5 mg/ml) was injected at the base of the tail in order to induce arthritis. The day (D) of arthritis induction was considered as D0. Rats were killed at D17, left ankles and blood samples were collected and stored at −80°C until further investigations.

### Nefopam Administration

The animals were divided into four groups. In the nefopam groups, rats received nefopam either IP at 20 mg/kg/day (*n* = 5) or orally at 60 mg/kg/day (expected dose) (8) in sugar water (1% sucrose) to mask nefopam bitterness (*n* = 5). In the two control groups, rats received NaCl 0.9% IP (*n* = 4) or oral sugar water (1% sucrose; *n* = 4). The nefopam administration began at D6, 4 days before arthritis onset at D10 in this model. Drink consumption was assessed 1 week before nefopam administration through the percentage of body weight. Then, drink consumption was measured every day to assess and adapt the nefopam dose received. Drink consumption was normalized by body weight to have an approximate idea of the nefopam dose received by animals.

### Clinical and Pain Monitoring

Clinical monitoring was performed by the measurement of two parameters representative of arthritis severity: arthritic index (AI) based on joint swelling and animal mobility and ankle circumference. As previously used (5), the AI was 0 for no change, 1 for small erythema, 2 for low to moderate erythema or swelling, 3 for pronounced edema with limited joint usage, and 4 for excess edema without joint usage. Ankle perimeter was estimated by antero-posterior and latero-lateral measurements with a caliper. The pain was assessed every day since D6 with scoring based on behavioral indicators such as the rat grimace scale (RGS), posture, locomotion, appearance, and temperament (10). Pain scoring is detailed in Supplementary Table 1. Body weight was measured every day since the beginning of the protocol.

### Histology

Right hind ankles were fixed in 10% formalin for 72 h at 4°C. After decalcification, ankles were cryoprotected, embedded, and frozen at −80°C. Cryosections (30 μm) were performed using Microm HM 560 Cryostat (Thermo Fisher Scientific, Waltham, MA, USA) and stained with H&E. The histology of ankle allowed us to assess inflammation especially in the navicular bone, which is the most sensitive bone in the AIA model (5).

### RNA Analysis

Left ankles were dissected under sterile conditions, frozen in liquid nitrogen, and then stored at −80°C until extraction. Tissue lysis was performed in TRI Reagent (Sigma, Saint Louis, MO, USA). RNA was separated from proteins after centrifugation in chloroform. RNA was purified with RNeasy plus (Qiagen, Venlo, Netherlands). Quality and quantity of RNA were assessed by the Experion RNA analysis (BioRad, Hercules, CA, USA) and QuantIT RiboGreen RNA assay (Thermo Fisher Scientifc), respectively. Reverse transcription (RT) was performed on 1 μg of RNA. Quantitative RT polymerase chain reaction (PCR) was conducted on the CFX96 RealTime System (BioRad) with LightCycler FastStart DNA Master plus SYBRgreen I (Roche Diagnostics, Basel, Switzerland). The results were normalized to the housekeeping gene expression hypoxanthine-guanine phosphoribosyltransferase. Primer sequences of candidate genes are detailed in Supplementary Table 2.

### Microcomputed Tomography

Rat ankles were scanned *ex vivo* with a microcomputed tomography (μ-CT; VivaCT40, Scanco, Brüttisellen, Switzerland) at 70 kVp (peak kilovoltage) and reconstructed under a resolution of 12.5 μm. Segmentation parameters for reconstruction and trabecular analysis were (sigma/support/threshold) 1/2/215 for trabecular bone and 0.7/2/215 for cortical bone.

### Statistical Analysis

All statistical analyses were performed using R software. A nonparametric analysis was performed by the Wilcoxon test. Results were considered significantly different when *p* < 0.05. When no difference was observed between animals that received the treatment IP and orally for each group, data were pooled as follows; the nefopam group includes all animals which received nefopam IP and orally, and the control group includes animals that received NaCl 0.9% IP and sugar water through drinks.

## Results

### No Impact of Nefopam on Arthritis Onset and Severity

**Figure 1:**
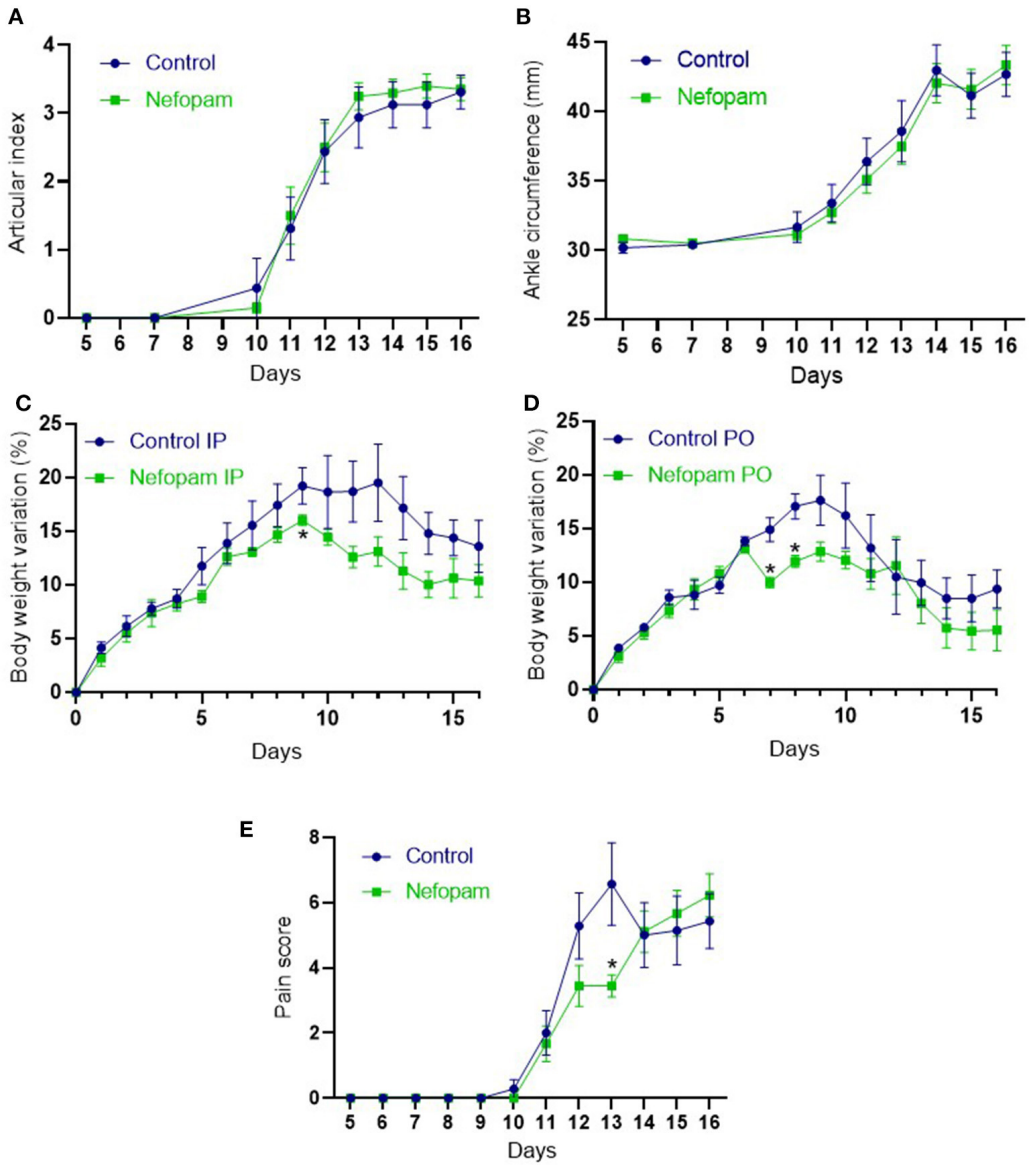
Clinical and pain monitoring during the rat adjuvant-induced arthritis (AIA). **(A)** Articular index (range from 0 to 4) from D5 to D16 in the control and nefopam groups. **(B)** Ankle circumference (representing the mean between the two ankles circumference) from D5 to D16 in the control and nefopam groups. **(C,D)** Body weight variation (%) from D0 to D16 in the control and nefopam groups which received treatment intraperitoneally **(C)** and orally **(D)**. **(E)** Pain score (range from 0 to 17) from D5 to D16 in the control and nefopam groups. The control group (in blue) included 8 AIA rats and the nefopam group (in green) included 10 AIA rats for each graph. Data are presented as mean ± standard error of the mean (SEM). The comparison between groups was performed at each time point by the Wilcoxon tests, *p < 0.05. IP, intraperitoneal; PO, *per os*; AIA, adjuvant-induced arthritis; D, days.

We first evaluated the effect of nefopam on arthritis severity. Arthritis onset was at the same time in all 4 groups. AI and mean ankle circumference began to increase at D10 for nefopam and control groups. Mean clinical score and mean ankle circumference kinetics were not different between nefopam and control groups (Figures 1A,B).

### Reduction of Pain by Nefopam in the Early Stage of Arthritis

In groups that received treatment IP, body weight variation from D0 was lower at D9 in the nefopam group *vs*. the control group, with a (*p* = 0.06) decreasing trend at D11, D13, and D14 (Figure 1C). In groups that received treatment orally, body weight variation was lower at D7 and D8 in the nefopam group before returning to a similar body weight variation than the control group (Figure 1D). Moreover, a lower drink intake was observed in the group taking nefopam orally compared to other groups, especially at D7 (data not shown). Rats that received more nefopam orally around 40–60 mg/kg/day were compared to those that consumed nefopam IP at 20 mg/kg/day (*p* < 0.05). Measures of pain indicators were similar in the nefopam and control groups for each day, except at the D13. At this time when the clinical kinetic score reaches its zenith, the nefopam group showed few pain indicators than the control group (Figure 1E).

### Small Impact of Nefopam on Arthritic Joint Inflammation

**Figure 2:**
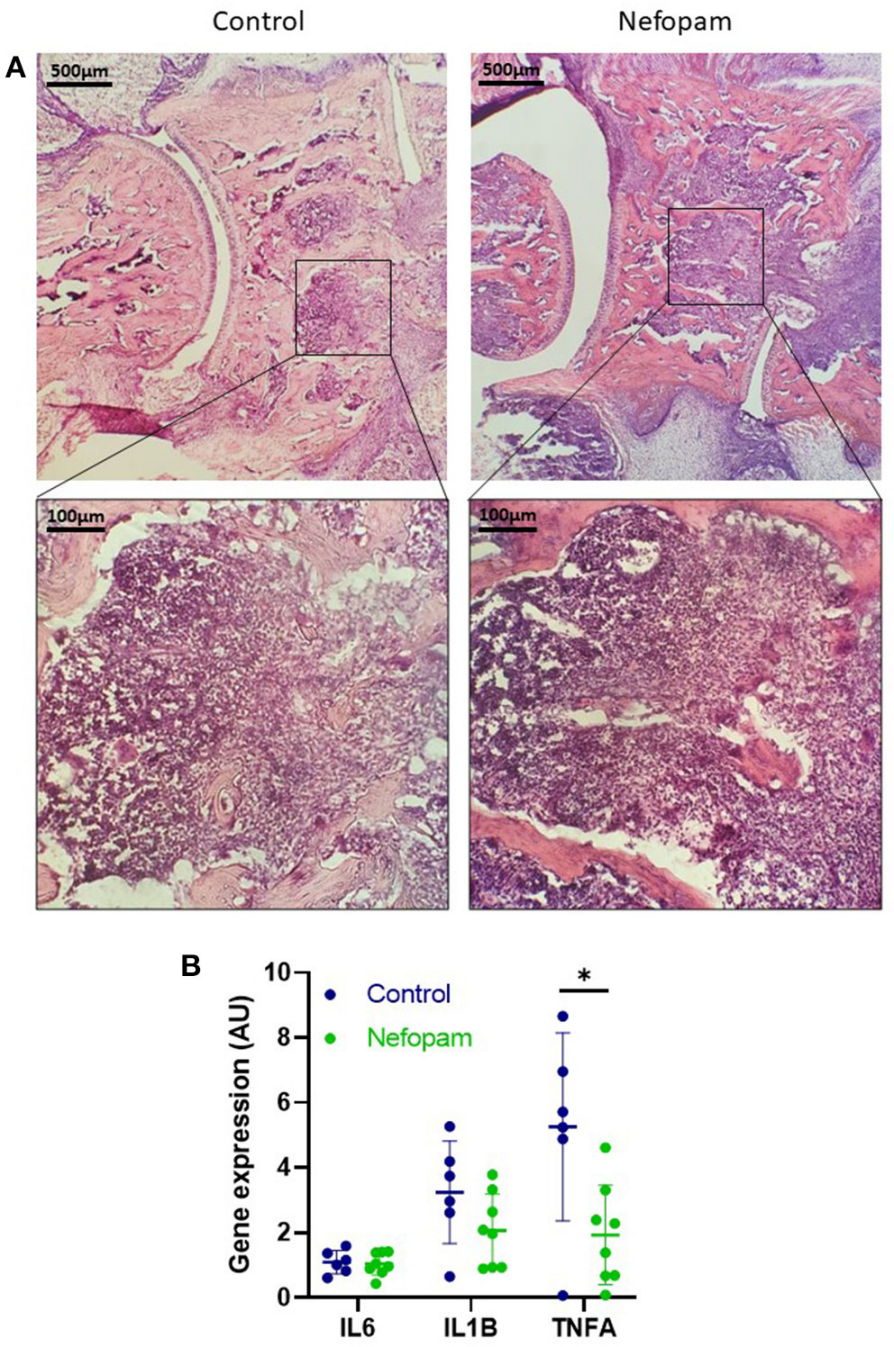
Joint inflammation at day 17 in the AIA rats. **(A)** Histological assessment of the astragal bone (on the left) and the navicular bone (on the right) with H&E staining on the frozen ankle section (30 μm). Representative slices were provided from rats at D17 (AI: 4) in the control and nefopam groups. Purple staining is mainly related to inflammatory cell infiltration. **(B)** Expression of inflammatory genes IL6, IL1B, and TNFA were normalized by the HPRT expression. The control group (in blue) included 6 AIA rats and the nefopam group (in green) included 8 AIA rats. Scatter dot plots are mean ± *SD*. Comparison between groups were performed by the Wilcoxon tests, *p < 0.05. AU, arbitrary units; AI, articular index; HPRT, housekeeping gene hypoxanthine-guanine phosphoribosyltransferase; AIA, adjuvant-induced arthritis; D, days.

Inflammation of the synovial membrane was similar in the nefopam and control groups, especially in the navicular bone with the infiltration of immune cells with synovial hypertrophy (Figure 2A). At the transcriptional levels, interleukin (IL) 6 and IL1B were expressed at a same level in the nefopam and control groups. However, tumor necrosis factor A (TNFA) expression decreased in the nefopam group *vs*. the control group (Figure 2B; *p* < 0.05).

### No Variation of Arthritic Joint Bone Loss Induced by Nefopam

**Figure 3:**
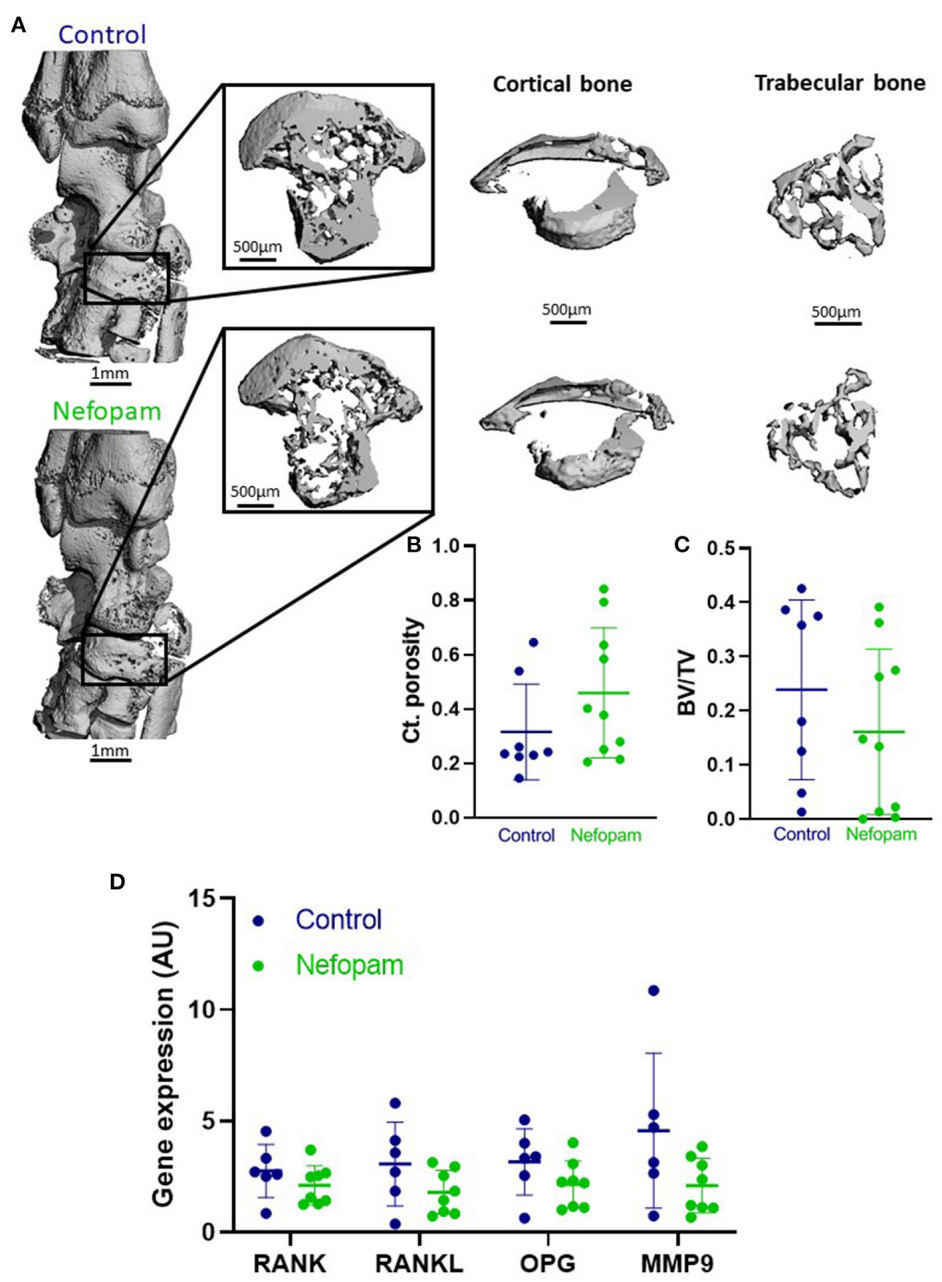
**(A)** Representative 3D μ-CT overall reconstruction of ankle and navicular bone (37 slices) for control (above) and nefopam (below) groups (AI: 4). **(B,C)** Cortical porosity (Ct. porosity) and bone volume/total volume (BV/TV) for control and nefopam groups. **(D)** Expression of osteoclastogenesis genes RANK, RANKL, OPG and MMP9 normalized by HPRT expression. Control group (in blue) included 6 AIA rats and nefopam group (in green) included 8 AIA rats. Scatter dot plots are mean ± SD. AU, arbitrary units. AI, articular index; HPRT, housekeeping gene hypoxanthine-guanine phosphoribosyltransferase; AIA, adjuvant-induced arthritis.

We evaluated the effect of nefopam administration on bone loss related to arthritis. In order to detect a potential difference in bone parameters, cortical and trabecular analyses were performed at the same architectural part of the navicular bone (37 slices; Figure 3A), which is already described to be the most sensitive to change among all ankle bones in arthritis evaluation (5). Even if there is an increasing trend (*p* = 0.1822 for cortical porosity) of bone loss in the nefopam group compared to the control group, cortical porosity and bone volume/total volume (BV/TV) were not different between the two groups (Figures 3B,C). These results were consistent with the gene expression of osteoclastogenesis and matrix degradation markers in the ankle. Indeed, receptor activator of nuclear factor-kappa B (RANK), RANK ligand (RANKL), osteoprotegerin (OPG), or matrix metalloproteinase (MMP) 9 were expressed at a same level in the nefopam and control groups (Figure 3D).

## Discussion

The first observation was that nefopam in a preventive approach did not interfere with disease onset and progression in the rat AIA model. Furthermore, in animals with high inflammation at the zenith of clinical scores (at D13), nefopam slightly reduced the pain score. Nefopam has been shown to have antinociceptive effects on rodents (8, 9), so its administration should decrease arthritis-induced pain perceived by animals and may also lead to a higher level of mobility. A previous study has demonstrated a correlation between nefopam administration and improvement of motor coordination (11). However, the increased mechanical loading on hind paws could increase pain in animals and would explain the lack of difference in pain assessment between nefopam and control groups. Besides, this hypothesis can be supported by an increasing trend in bone loss in the nefopam group, where cortical porosity showed an increasing trend and trabecular volume a decreasing trend compared to the control group. As a result, an increased joint inflammation in the nefopam group could be expected. The synovial hypertrophy was similar to immune cell infiltration in both groups, suggesting that nefopam did not reduce joint inflammation. However, TNFA expression was surprisingly lower at the transcriptional level in the nefopam group compared to the control group despite the increasing trend of bone loss. Nefopam has proven to inhibit TNF production in the spinal cord (12). Thus, we could explain the lower level of this proinflammatory cytokine in the nefopam group. However, others cytokines were similarly expressed in both groups; and arthritis-induced bone loss was not altered by the lower level of TNFA, as TNF plays a major role in MMP production and osteoclastogenesis stimulation (13).

In our study, the AIA model was used due to its reliability in onset and progression (4). Furthermore, compared to other induced arthritis models like a collagen-induced model with the incidence of arthritis as around 30–90% (14), the AIA model reaches a high incidence of arthritis (90–100%) (4). To assess pain, a pain score based on behavioral physiologic changes was used to avoid pain related to stress handling (15). Moreover, the RGS was already described as a reliable method to quantify spontaneous pain in rat (16). At the peak stage of pain, nefopam was effective at D13 compared to control. Body weight is also considered as a good indirect indicator of pain (15). However, in this situation, weight was not a good candidate since nefopam can induce some bitterness. In fact, nefopam IP showed a downward trend in the body weight compared to the control group since the beginning of nefopam treatment. Nefopam injected IP showed positive association toward weight loss whereas nefopam when taken orally prevented weight gain only in the first 2 days, after which nefopam was given orally in sucrose water to which rats acclimatized better. The discrepancy in weight loss could be attributed to pain induced by IP or impact of metabolism.

It is important to mention some limitations in this study. First of all, the number of animals per group was not very high but was sufficient to perform statistical analyses. This choice was made from an ethical point of view in order to reduce the number of animals that could suffer. For the animals that received oral nefopam, the dose per animal was not exactly known since the animals were housed in groups in order to avoid social stress. However, this mode of administration is not invasive and depends on the voluntary activity of the animals. It could also be hypothesized that oral administration ensures a continuous effect on pain management unlike IP administration, which can be intermittent.

To conclude, nefopam was effective in reducing pain in the AIA model in the early stage of the arthritis. Oral administration could be the best way to treat pain in this model as it is less invasive than other administration methods, and nefopam dosage can be easily adapted in order to reduce pain score. However, its administration after the pain peak should be reconsidered as the pain was no longer reduced. Moreover, a prolonged administration of nefopam slightly reduced the inflammation. More investigations are necessary to determine the best administration protocol of nefopam in the AIA model, in order to apply it on other inflammation disease models and then improve pain management.

## Data Availability Statement

The original contributions presented in the study are included in the article/Supplementary Materials, further inquiries can be directed to the corresponding author.

## Ethics Statement

The animal study was reviewed and approved by Ethical Committee for Animal Experiments of Saint-Etienne University (agreement number: 2019032816186046).

## Author Contributions

ED and HM designed the study, analyzed the results, and wrote the manuscript. ED, HM, MM, AV-B, and SP contributed to the *in vivo* experiment. ED and M-TL carried out molecular biology. All authors approved the final version.

## Funding

Medac provided nefopam for free for this research.

## Conflict of Interest

HM reports non-financial support from Medac related to this work. Medac provided for free the nefopam. HM reports personal fees and non-financial support from AbbVie; personal fees from Accord; personal fees from Amgen; grants, personal fees and non-financial support from Biogen; grants, personal fees, and non-financial support from Bristol Myers Squibb; personal fees and non-financial support from Cell Trion Healthcare; grants and non-financial support from Fresenius Kabi; personal fees and non-financial support from Galapagos; non-financial support from Janssen; grants personal fees, and non-financial support from Lilly; personal fees and non-financial support from MSD; grants, personal fees, and non-financial support from Nordic Pharma; personal fees, non-financial support and other from Novartis; grants, personal fees and non-financial support from Pfizer; personal fees from Roche Chugai; grants, personal fees, and non-financial support from Sanofi, outside the submitted work. The remaining authors declare that the research was conducted in the absence of any commercial or financial relationships that could be construed as a potential conflict of interest.

## Publisher's Note

All claims expressed in this article are solely those of the authors and do not necessarily represent those of their affiliated organizations, or those of the publisher, the editors and the reviewers. Any product that may be evaluated in this article, or claim that may be made by its manufacturer, is not guaranteed or endorsed by the publisher.
