# Supplementary material for: Improvement of Pain Management by Nefopam in a Rat Adjuvant-Induced Arthritis Model
Source: Front Vet Sci. 2022 Apr 26;9:809980. doi: 10.3389/fvets.2022.809980 (PMC9087999; doi:10.3389/fvets.2022.809980)
Supplement: Supplementary file 1 [file Data_Sheet_1.docx]

**Table S1**: **Pain scoring**

| **Rat grimace scale** | |
| --- | --- |
| Orbital tightening | 0-2 |
| Nose/Cheek flattening | 0-2 |
| Ear changes | 0-2 |
| Whisker changes | 0-2 |
|  |  |
| **Posture and mobility** | |
| Normal posture | 0 |
| Huddling | 1 |
| Normal mobility | 0 |
| Low mobility | 1 |
| No mobility | 2 |
|  |  |
| **Peeling** | |
| Clean peeling | 0 |
| Dirty peeling | 1 |
| Pitted hair | 1 |
|  |  |
| **Temperament** | |
| Decrease exploratory behavior | 1 |
| Lethargy | 1 |
| Isolation | 1 |
| Aggressive behavior | 1 |

**Table S2**: **PCR primer sequences in 5’-3’ direction**

| Target | Species | Primers | Tm (°C) | Product size (bp) | Source |
| --- | --- | --- | --- | --- | --- |
| IL6 | Rat | Forward: ttccagccagttgccttctt  Reverse: cagtgcatcatcgctgttca | 63.0 | 225 | NM_012589.1 |
| IL1B | Rat | Forward: gcttccttgtgcaagtgtct  Reverse: aagctggatgctctcatctg | 63.0 | 176 | NM_031512.2 |
| TNFA | Rat | Forward: cacgctcttctgtctactga  Reverse: gtaccaccagttggttgtct | 63.0 | 254 | NM_ 012675.3 |
| RANK | Rat | Forward: atcgtcctgctcctcttcat  Reverse: acttcttgctggctggagtt | 64.0 | 200 | NM_001271235.1 |
| RANKL | Rat | Forward: gacagcacgcgctgcttcta  Reverse: ccacatcgagccacgaacct | 63.0 | 220 | NM_057149.1 |
| OPG | Rat | Forward: gagtgtgcgaatgtgaggaa  Reverse: aattagcaggaggccaagtg | 63.0 | 215 | NM_012870.2 |
| MMP-9 | Rat | Forward: ttcgacgacgacgagttgtg  Reverse: tgccatgctccgtgtagaga | 63.0 | 230 | NM_031055.1 |
